# Prostaglandin E_2_ stimulates progression-related gene expression in early colorectal adenoma cells

**DOI:** 10.1038/sj.bjc.6603146

**Published:** 2006-05-09

**Authors:** I Mauritz, S Westermayer, B Marian, N Erlach, M Grusch, K Holzmann

**Affiliations:** 1 Department of Internal Medicine 1, Institute of Cancer Research, Medical University Vienna, Borschkegasse 8a, A1090 Vienna, Austria

**Keywords:** colon carcinogenesis, adenomatous polyps, prostaglandins, cyclooxygenase-2, PG-dependent gene expression

## Abstract

Upregulation of cyclooxygenase-2 (COX-2) and prostaglandin-dependent vascularisation in small adenomatous polyps is an essential part of colon carcinogenesis. To study the underlying cellular mechanisms, LT97 and Caco2 human colorectal tumour cells not expressing endogenous COX-2 were exposed to 1 *μ*M prostaglandin E_2_ (PGE_2_) in their medium. At 30 min after addition, expression of c-fos was stimulated 5-fold and 1.3-fold, respectively, depending on the activation of both extracellular signal-regulated kinase and p38. The amount of c-jun in nuclear extracts was increased 20% in LT97 cells. Expression of COX-2 was upregulated 1.7-fold in LT97 cells and 1.5-fold in Caco2 2 h after prostaglandin (PG) addition by a p38-mediated pathway. The known PGE_2_ target gene vascular endothelial growth factor (VEGF) was not modulated. Effects of sustained PGE_2_ production were studied in VACO235 cells that have high endogenous COX-2 and in LT97 cells infected with an adenovirus expressing COX-2. Prostaglandin E_2_ secretion into the medium was 1–2 nM and 250 pM, respectively. Expression of both VEGF and c-fos was high in VACO235 cells. In LT97 cells, COX-2 upregulated c-fos expression and c-jun content in nuclear extracts 1.7- and 1.2-fold, respectively, in a PG-dependent way. This shows that exogenous PGE_2_ as well as COX-2 overexpression affect signalling and gene expression in a way that enhances tumour progression.

Upregulation of cyclooxygenase-2 (COX-2) expression in early adenomas is an essential prerequisite of colorectal carcinogenesis. It causes elevated levels of prostaglandins (PGs) in the tissue and PG-dependent neovascularisation (Eberhart *et al*, 1994). Adenomatous polyps regress under therapy with non-steroidal anti-inflammatory drugs whose main cellular target is COX-2 (DuBois *et al*, 1996). Mice carrying a hereditary mutation in the APC gene (min-mice or transgenic ΔAPC-mice; (de Wind *et al*, 1998) develop a multitude of intestinal polyps in a COX-2 and prostaglandin E_2_ (PGE_2_)-receptor-dependent way (Sonoshita *et al*, 2001; Seno *et al*, 2002; Sunayama *et al*, 2002). Immunohistochemical analysis of tissue sections shows that much of the COX-2 is located in connective tissue but not in the epithelial compartment of the polyps. In the pathology of human colorectal cancer, COX-2 plays a similar role and has become the main target of chemoprevention in high-risk patients (DuBois *et al*, 1996; Gupta and Dubois, 2001). As in the mouse models, COX-2 is mainly located in the connective tissue of human adenomatous polyps.

This raises the question whether PGs produced in the microenvironment (fibroblasts, endothelial cells or macrophages) can in turn affect early premalignant cells and trigger expression of tumour-associated genes. To answer these questions we have established the human colonic adenoma cell line LT97 from microadenomas of a polyposis patient that do not yet express COX-2 or produce PGE_2_ (Richter *et al*, 2001). Tumour-promoting bile acids have been shown to induce expression of c-fos, COX-2 and vascular endothelial growth factor (VEGF) in these early human colonic adenoma cells (Jurek *et al*, 2005). The current study analysed the following: (1) the cellular effects of exogenous PGE_2_ on the expression of COX-2, c-jun and c-fos in LT97 human colorectal adenoma cells as well as Caco2 colorectal carcinoma cells that also express little COX-2, (2) the consequences of sustained COX-2 expression in VACO235 cells that have a high level of endogenous expression of the enzyme (Willson *et al*, 1987) and in LT97 cells infected with a COX-2-expressing adenovirus.

## MATERIALS AND METHODS

### Tissue culture and exposure to PG

LT97 colon adenoma cells were established by our laboratory (Richter *et al*, 2002). They were kept under standard tissue culture conditions by using Ham F-12 medium containing 20% L-15 medium, 2% FCS, 10 *μ*g ml^−1^ insulin, 2 × 10^−10^ M triiodotyronine_,_ 2 *μ*g ml^−1^ transferrin, 1 *μ*g ml^−1^ hydrocortisone, 5 × 10^−9^ M Na-selenite and 30ng ml^−1^ epidermal growth factor (EGF). Under these conditions, LT97 cells have a doubling time of ∼96 h. VACO235 adenoma cells were a gift from Willson *et al* (1987) and were cultured with minimal essential medium (MEM) supplemented with 2% FCS, 10 *μ*g ml^−1^ insulin, 2 × 10^-10^ M triiodotyronine, 2 *μ*g ml^−1^ transferrin, 1 *μ*g ml^−1^ hydrocortisone, 5 × 10^−9^ M Na-selenite and 30 ng ml^−1^ EGF. Their doubling time was ∼60 h. Caco2 colorectal carcinoma cells were obtained from the American Type Culture Collection and propagated in MEM containing 10% FCS with a doubling time of about 48 h. HEK293 cells were a gift of Dr M Herlyn (Wistar Institute). They were cultivated in RPMI medium containing 10% FCS.

Prostaglandin E_2_ or vehicle was added to LT97 cells 96 h, and to Caco2 cells 48 h, after plating. For inhibition of COX-2, SC236 (Searle Skokie, IL, USA) was added concomitantly with the PG in a concentration of 1 *μ*M that did not affect proliferation of LT97 cells. The kinase inhibitors U0126 and PD169316 were purchased from Calbiochem (San Diego, CA, USA)

### Cell number

The cell number was determined by neutral red uptake during a period of 2 h from serum-free MEM containing 50 *μ*g ml^−1^ of the dye, which was taken up into the lysosomes of viable cells. After removal of excess dye by washing with PBS, neutral red taken up into the cells was extracted with 1% acetic acid in 70% ethanol. Absorbance was measured at 562 nm using a plate reader.

### Construction of adenoviral vectors expressing COX-2

The entire coding region of human COX-2 was obtained by amplification with PFU DNA polymerase (Stratagene, La Jolla, CA, USA) using primers 5′-TAAGTCGACCGCTCGGATGCTCG-3′ and 5′- GACTCTAGACTACAGTTCAGTCGAACG-3′ from the cDNA of EST clone CS0DK012YH07 (Invitrogen, CA, USA) from a human cervix carcinoma cell line. The PCR product was first cloned into pCR2.1-TOPO vector (Invitrogen) and further transferred via restriction with *Eco*RI into the expression vector pCMV-SPORT6 (Invitrogen) in sense orientation. The recombinant protein from the sense construct was expressed *in vitro* from the SP6 promoter as a 70 kDa protein using the TNT-SP6 Coupled Reticulocyte Lysate System (Promega, Mannheim, Germany). The cDNA fragments were further subcloned with the restriction enzymes *Hind*III and *Kpn*I into the pShuttle-CMV (Stratagene).

The adenoviral expression vector Ad-COX-2 was created by a double-recombination event in bacteria between cotransformed adenoviral plasmid pAdEasy-1 (Stratagene) and shuttle vector pShuttle-CMV based on the method described by He *et al* (1998). Briefly, the shuttle vector was linearised with *Pme*I, gel purified and cotransformed into BJ5183 cells by electroporation. Single clones were selected and confirmed by *Pac*I digestion of plasmid DNA. Plasmids from correct clones were amplified by transformation of XL10 cells (Stratagene) followed by DNA maxiprep (Qiagen, Helden, Germany). The adenoviral DNA of Ad-COX-2 was linearised with *Pac*I, heat-inactivated, ethanol-precipitated and used for transfection of the packaging cell line HEK293 with Lipofect2000 (Invitrogen). Shuttle vector without any insert was used to construct control virus (Ad-co). Transfected cells were incubated for 7–10 days until cytopathic effect appeared. Primary virus was harvested, amplified and purified using a double CsCl gradient. Virus titer was determined by a standard plaque assay using HEK293 cells.

### Determination of gene expression

Total RNA was isolated with TRIZOL (Gibco Life Technologies, Paisley, UK) according to the manufacturer's instructions. RNA (2 *μ*g) was used to synthesise cDNA by reverse transcription using random hexamer primers and M-MLV reverse transcriptase (Sigma, St Louis, MO, USA).

Genes of interest were amplified from cDNA samples by standard PCR cycles of 1 min denaturation (94°C), 30 s annealing and 1 min synthesis at 72°C. Reaction products were separated on 6% polyacrylamide gels and stained with 0.5 *μ*g ml^−1^ ethidiumbromide. For quantitation, GelDoc 2000 system and the software Quantity One 4.2.1. (Bio-Rad Laboratories, USA) were used.

Linearity of amplification was determined at the range of 25–35 cycles for target genes and 18–25 cycles for GAPDH to chose the adequate number of cycles for each analysis. For the number of cycles chosen, dose dependency was confirmed by using increasing amounts of cDNA. Primer sequences, annealing temperatures and number of cycles are listed in Table 1.

### Quantification of gene expression by real-time PCR

c-fos, COX-2 and VEGF were amplified from cDNA samples by real-time PCR using TaqMan assays on an ABI PRISM 7000 Thermocycler (Applied Biosystems, Foster City, CA, USA). After denaturation for 10 min at 95°C, 40 cycles of 15 s denaturation (95°C) and 60 s annealing and synthesis at 60°C were performed using TaqMan Assay-on-Demand kits (Table 2) and TaqMan Universal PCR Master Mix. Quantitative assessment was performed using the ΔΔ*C*_T_ method.

### Western blotting

Cells were dislodged from the culture plate by using a cell scaper, collected by centrifugation and washed 2 × with icecold PBS. Cell pellets were homogenised in RIPA-buffer (50 mM Tris/HCl pH 7.4; 500 mM NaCl, 1% NP-40, 0.5% Na-DOC, 0.1% SDS, 0.05% NaN_3_, 20 *μ*l ml^−1^ Complete Protease Inhibitor (Roche Diagnostics, Vienna, Austria), 500 *μ*M sodium vanadate and 5 mM sodium fluoride). The insoluble fraction was removed by centrifugation for 15 min at 4°C and 14 500 r.p.m. A total of 20 *μ*g protein per lane were analysed by electrophoresis on 12 or 15% polyacrylamide gels and transfered to PVDF-membranes. Membranes were probed with antibodies directed against extracellular signal-regulated kinase (ERK) (1 : 10 000; Upstate Biotechnology, Lake Placid, NY, USA) and p-ERK (1 : 5000; Sigma, St Louis, MO, USA), p38 (1 : 1000; Santa Cruz, Santa Cruz, CA, USA) and p-p38 (1 : 1000; Sigma) and COX-2 (1 : 200; Transduction Laboratories). Secondary horseradish-peroxidase-coupled antibodies were diluted 1 : 5000 and detected by the Super Signal West Pico Western blotting detection system (Pierce, Rockford, IL, USA).

### DNA-binding c-jun in the nucleus

DNA-binding c-jun was determined from nuclear extracts using the Mercury Transfector kit (Clontech, Palo Alto, CA, USA) according to the manufacturer's instructions. In short, nuclei were isolated from about 1–5 × 10^7^ cells lysed in 10 mM HEPES pH 7.9 containing 1.5 mM MgCl_2_, 10 mM KCl, 10 mM DTT and 0.1 *μ*l ml^−1^ Complete Protease-Inhibitor (Roche). Nuclei were extracted using 20 mM HEPES pH 7.9 containing 1.5 mM MgCl_2_, 0.42 M NaCl, 0.2 mM EDTA, 10 mM DTT, 0.1 *μ*l ml^−1^ Complete Protease-Inhibitor and 25% (v v^−1^) glycerol. A total of 30 *μ*g extracted protein was bound to 96-well plates coated with c-jun-binding oligonucleotide. After washing the plates and blocking of unspecific binding sites, c-jun was detected using an ELISA detection reaction.

### PG production

Cells were left to secrete PGE_2_ for 24 h after addition of 10 *μ*M arachidonic acid substrate into the medium. Prostaglandin E_2_ secreted during this period was determined using an indirect ELISA kit purchased from Caymen Chemicals (Ann Arbor, MI, USA) according to the manufacturer's instructions.

## RESULTS

### Stimulation of cell growth by PGE_2_

**Figure 1:**
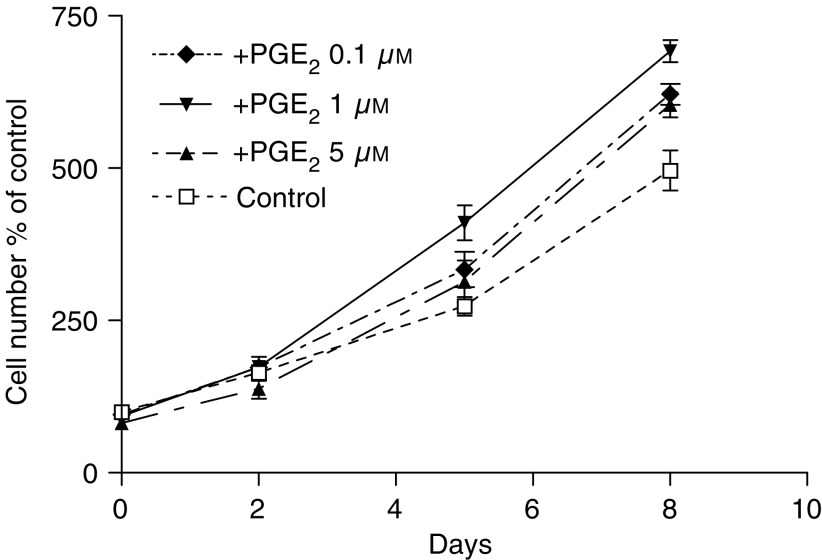
Stimulation of adenoma cell growth by PGE_2_. A measure of 0.1, 1 and 5 *μ*M PGE_2_ was added to the culture medium 2 days after plating and cell number determined 2, 5 and 8 days later.

Two days after seeding, increasing concentrations of PGE_2_ were added to the culture medium of LT97 human colorectal adenoma cells and cell number was determined at days 2, 5 and 8. Growth was stimulated by all PG concentrations, with the optimal concentration at 1 *μ*M (Figure 1). Consequently, the 1 *μ*M concentration was used for most experiments elucidating signalling events and gene expression.

### Modulation of COX-2 and VEGF gene expression by PGE_2_

**Figure 2:**
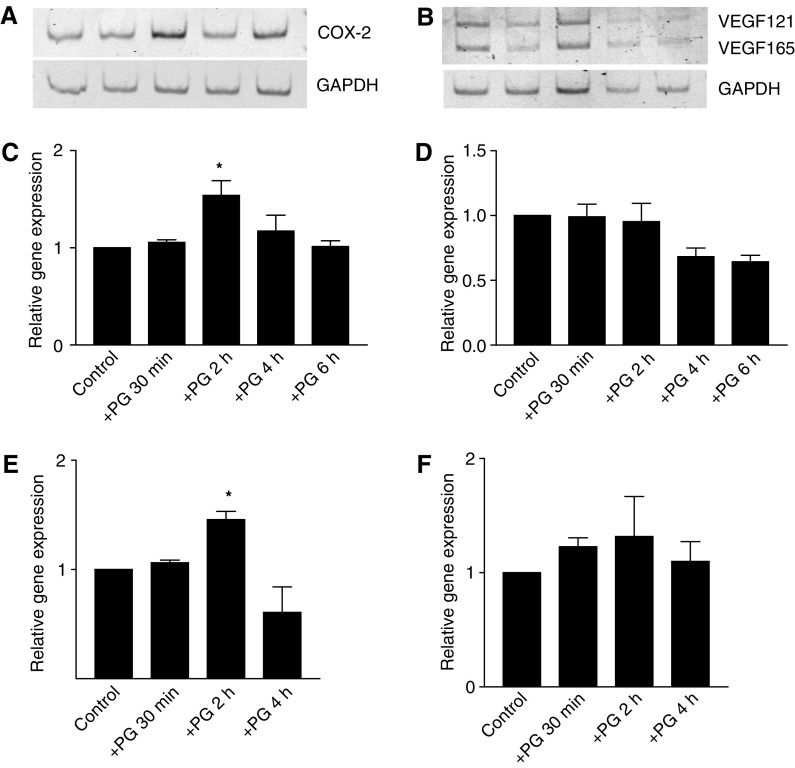
Expression of COX-2 and VEGF in LT97 adenoma and Caco2 carcinima cells. LT97 cells were exposed to 1 *μ*M PGE_2_ and transcripts were analysed after 30 min, 2, 4, and 6 h. Expression of COX-2 (**A**) and VEGF (**B**) was determined by RT–PCR and PCR products were analysed on 6% polyacrylamide gels. Quantification of gene expression was obtained by real-time PCR and calculated relative to GAPDH. Values given represent mean±s.e.m. from three independent experiments. (**C**, **D**) LT97 colorectal adenoma cells, (**E**, **F**) Caco2 colorectal carcinoma cells, (**C**, **E**) COX-2 and (**D**, **F**) VEGF. ^*^Increased above control at *P*<0.05.

To determine whether exogenous PGs can stimulate COX-2 expression and consequently their own production, LT97 cells as well as Caco2 colorectal carcinoma cells were exposed to 1 *μ*M PGE_2_ for up to 6 h and expression of COX-2 was determined by RT–PCR. Expression was elevated 2 h after PGE_2_ addition (Figure 2A), indicating the possibility that a positive feed-back loop of PG-production is established. Similar but smaller effects were obtained after addition of 5 *μ*M PGE_2_ (data not shown). In addition, the expression of VEGF in LT97 cells was analysed from the same RNAs. From five known splice variants, we could detect two main forms – VEGF165 and VEGF121; however, no changes in expression were observed owing to exposure to PGE_2_ (Figure 2B).

Real-time PCR was performed to obtain reliable quantification using cDNAs of the 1 *μ*M PGE_2_ groups (Figure 2C and D). The results showed a 50% increase of COX-2 expression (Figure 2C, *P*<0.05). Vascular endothelial growth factor expression was not increased but rather tendentiously suppressed 4 and 6 h after addition of PGE_2_ (Figure 2D).

Effects of PGE_2_ exposure were also measured in Caco2 colorectal carcinoma cells with similar results: expression of COX-2 increased 28% (*P*⩽0.05) 2 h after PG addition (Figure 2E), whereas expression of VEGF was variable and not significantly increased (Figure 2F).

### Effects on cellular signalling

**Figure 3:**
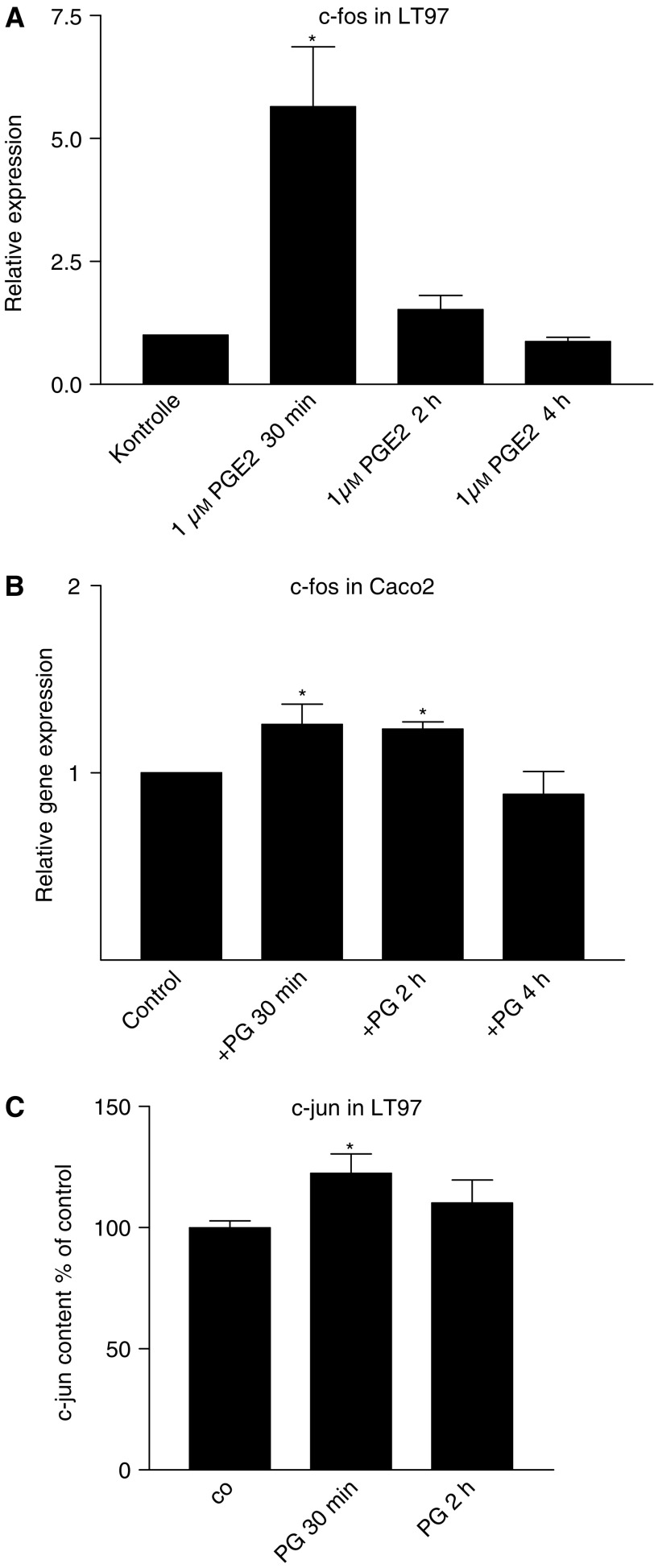
Induction of c-fos and c-jun by PGE_2_. Expression of c-fos was determined from the same cDNA preparation used in Figure 2 and quantified relative to GAPDH (**A**) LT97 cells, (**B**) Caco2 cells. c-jun protein was quantified from nuclear extracts obtained from LT97 cells by using an ELISA format detecting DNA-binding of c-jun (**C**). Values given represent mean±s.e.m. from three independent experiments. ^*^Increased above control at *P*<0.05.

Expression of c-fos was determined from RNAs isolated after exposure to PGE_2_ as described above using quantitative real-time PCR. Expression was stimulated about 6-fold 30 min after addition of the PGE_2_ (1 *μ*M) after which it rapidly returned to control levels (Figure 3A). In Caco2 cells c-fos induction by the PG was smaller. We did, however, observe significant increases both 30 min and 2 h after addition of PGE_2_ (Figure 3B).

Induction of c-jun was analysed from nuclear extracts of LT97 adenoma cells using c-jun-binding oligonucleotides and a specific c-jun antibody in an ELISA format. Addition of PGE_2_ to the cultures stimulated c-jun binding to the promoter sequence by about 20% (*P*<0.05; Figure 3C).

**Figure 4:**
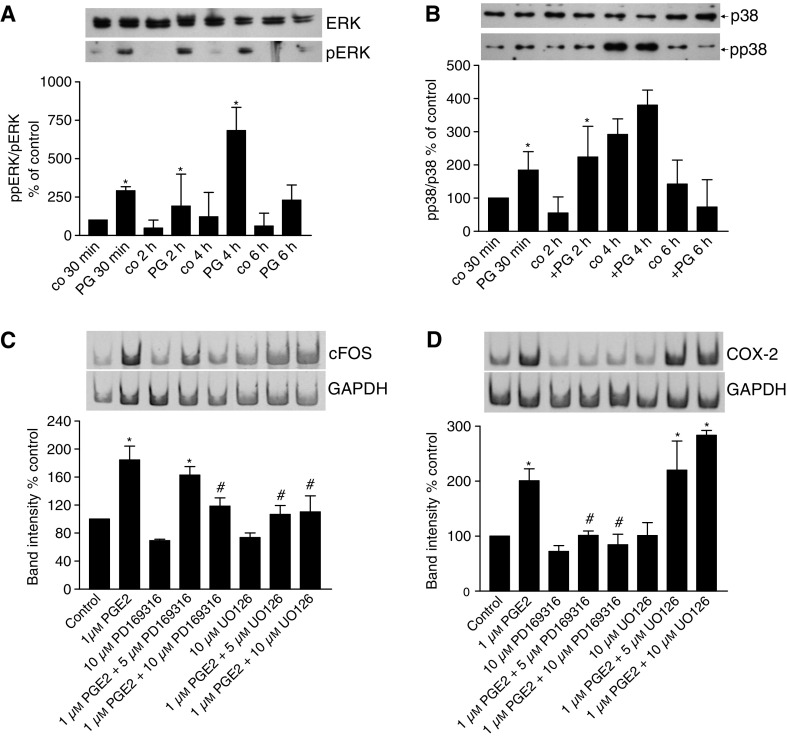
Activation of signalling kinases. Activation of the signalling kinases ERK (**A**) and p38 (**B**) in LT97 adenoma cells was analysed by Western blotting with phosphorylation-specific antibodies. Band intensities were semi-quantified using the Image Quant software. The results represent the mean±s.e.m. from three independent experiments. ^*^Larger than control at the same time point at *P*<0.05. U0126 or PD169316 were added to the culture medium concomitantly with the PG to inhibit phosphorylation of ERK and p38, respectively. RNA was isolated after 30 min and expression of c-fos (**C**) and COX-2 (**D**) was analysed by standard RT–PCR. Band intensities were semi-quantified using the Image Quant software. The results represent the mean±s.e.m. from three independent experiments. ^*^Larger than control at the same time point at *P*<0.05, # smaller than PG group at *P*<0.05.

For analysis of signalling kinases, LT97 cells were homogenised in RIPA buffer after 0.5, 2 and 4 h of exposure to 1 *μ*M PGE_2_. Proteins were separated by SDS–PAGE and phosphorylation of ERK and p38 was analysed using phosphorylation-specific antibodies. We observed increased phosphorylation of both signalling kinases (representative Western blot shown in Figure 4A and B). Semi-quantification of band intensities from four independent experiments demonstrates a 2–7-fold increase of ERK phosphorylation from 30 min to 4 h after PGE_2_ addition (Figure 4A). Phosphorylation of p38 was increased about 2-fold 30 min and 2 h after PGE_2_ addition. At the 4 h time point, phosphorylation in the control group had reached a level similar to the PGE_2_ group (Figure 4B).

To investigate whether the activation of ERK was essential for the induction of COX-2 and c-fos expression, the mitogen-activated protein kinase inhibitor U0126 was added to the culture medium together with the PG and COX-2 and c-fos expression assessed by standard RT–PCR. The inhibitor prevented induction of c-fos expression, whereas it did not affect expression of COX-2 (Figure 4C). Addition of PD169316, an inhibitor of p38 signalling, blocked expression of COX-2 as well as c-fos (Figure 4D).

### Effects of sustained PGE_2_ production in adenoma cell cultures

**Figure 5:**
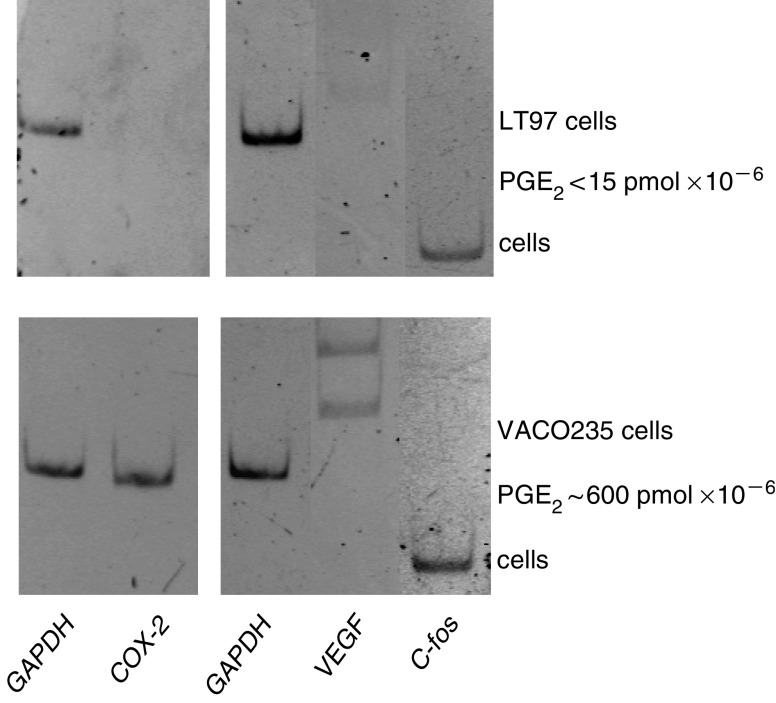
Gene expression pattern in adenoma cells. RNA was isolated from VACO235 and LT97 adenoma cell cultures at about 50% confluency. Expression of COX-2, VEGF and c-fos was determined by RT–PCR. Expression of GAPDH was determined for standardisation. Polymerase chain reaction products were analysed on 6% acrylamide gels. The figure shows a composite of two individual analyses each with its separate GAPDH control.

The data described above indicate that PGE_2_ present in the microenvironment is capable of triggering COX-2 expression and consequently sustained PG production by the adenoma cells themselves that potentially have even more pronounced effects on the expression of tumour progression-associated genes. VACO235 are villous adenoma cells that highly express COX-2 and secrete PGE_2_ up to a concentration of 1–2 nM into their culture supernatant (Willson *et al*, 1987; Richter *et al*, 2001). Expression of VEGF and c-fos in these cells was analysed by RT–PCR and found to be higher than that of LT97 cells (Figure 5).

**Figure 6:**
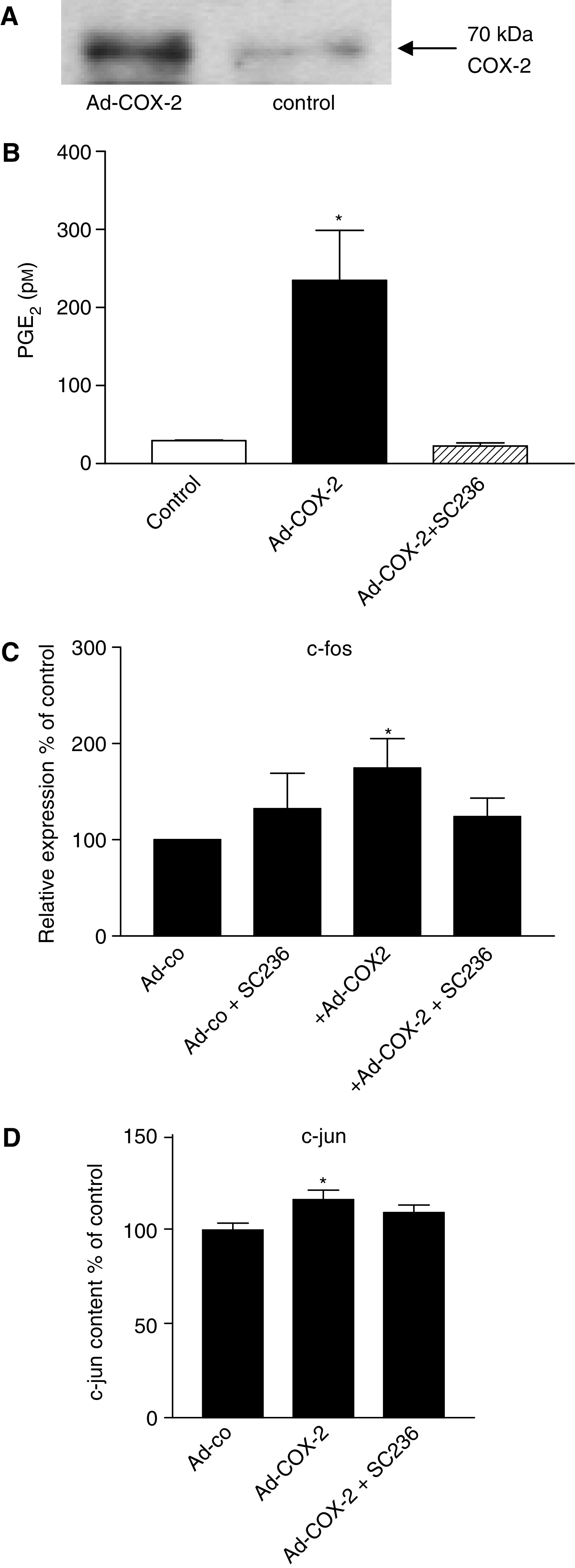
Gene expression in LT97 adenoma cells overexpressing COX-2. LT97 cells were infected with an adenoviral vector expressing COX-2. Control groups were infected with control viruses containing no ectopic gene and cells exposed to the COX-2 inhibitor SC236. (**A**) Cells were homogenised in RIPA buffer and COX-2 protein analysed by Western blotting. (**B**) Prostaglandin E_2_ secretion into the medium was determined by ELISA. (**C**) RNA was isolated from control transfectants and COX-2-LT97 cells in the presence and absence of the COX-2 inhibitor SC236 and c-fos expression was analysed by standard RT–PCR. Polymerase chain reaction products were seperated on 6% acrylamide gels and band intensities were semi-quantified using the Image Quant software. The results represent the mean±s.e.m. from three independent experiments. ^*^Increased above control at *P*<0.05. (**D**) c-jun in nuclear extracts from control cultures and COX-2-LT97 cells in the presence and absence of SC236 was quantified as described in Figure 2.

To obtain cells with high or low COX-2 expression in the same genetic background, we used an adenoviral vector expressing COX-2 under the control of a CMV promoter. LT97 cells were infected with 10 multiplicity of infection of the virus. At 24 h after infection, COX-2 protein could be detected in the infected cells (Figure 6A) and PG secretion into the medium increased from <15 pM to about 250 pM PGE_2_ (Figure 6B). Prostaglandin production could be completely inhibited by the specific COX-2 inhibitor SC236 (Figure 6B). Cell growth was not affected by the transfection (data not shown).

We then proceeded to analyse gene expression and signalling kinases in LT97 adenoma cells carrying a COX-2 adenovirus (LT97-COX-2 cells) in the presence and absence of SC236 and consequently, in the absence and presence of PG. Expression of the COX-2 target gene VEGF was highly variable as shown by the large standard deviation (Table 3). Expression of c-fos on the other hand was increased 1.7-fold in a PGE_2_-dependent manner (Figure 6C). Increased binding of c-jun from nuclear extracts to promoter DNA was induced about 20% (*P*⩽0.05, Figure 6D).

**Figure 7:**
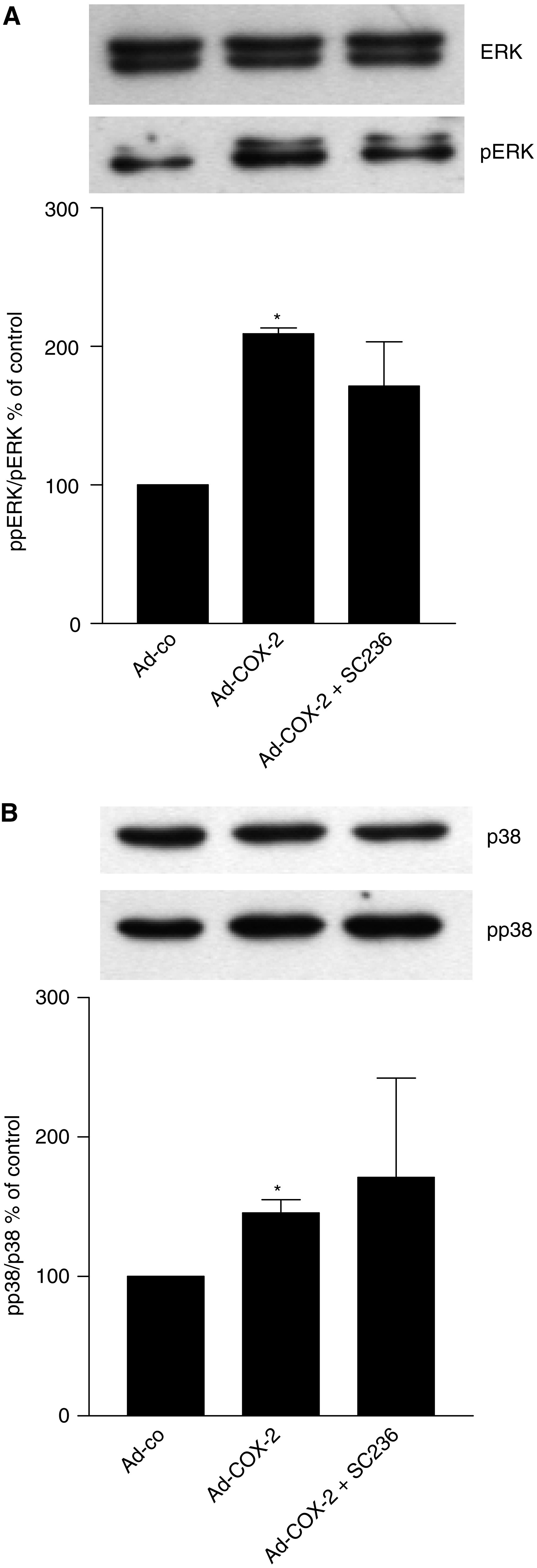
Activation of signalling kinases in COX-2-overexpressing cells. Activation of ERK and p38 was analysed from protein lysates from control cells as well as COX-2-LT97 cells in the presence and absence of SC236. Band intensities were semi-quantified using the Image Quant software. The results represent the mean±s.e.m. from three independent experiments. ^*^Larger than control at *P*<0.05.

Activation of ERK and p38 signalling was analysed by Western blot as described above and both kinases were activated in cells infected with the COX-2 virus. Although ERK phosphorylation was stimulated in a PG-dependent manner, phosphorylation of p38 in cells infected with the COX-2 virus was not affected by the COX inhibitor (Figure 7A and B).

## DISCUSSION

Histological examination of polyps in the min-mouse model suggested that COX-2 expression and PG production takes place primarily in the connective tissue. Sonoshita *et al* (2002) have identified fibroblasts and endothelial cells as the main source, whereas elevated COX-2 protein in the epithelial compartment was not observed. It has to be assumed therefore that at this early stage of tumour development, adenoma cells are subject to elevated levels of prostaglandin produced locally in the tumour microenvironment. This paper has investigated the effects this may have on tumour progression.

As cell line models, we have used LT97 human colorectal adenoma cells and Caco2 colorectal carcinoma cells that express only minimal amounts of COX-2 and produce little PGE_2_ (Richter *et al*, 2001). Exposure to PGE_2_ stimulated growth of the adenoma cells with an optimal concentration of 1 *μ*M as well as in the Caco2 cells (Pai *et al*, 2002). As compared to the amounts of PG secreted by COX-2-expressing cells into their culture medium, this concentration is very high, which may explain why smaller effects were observed with the highest concentration (5 *μ*M). A similar tendency to inhibit proliferation has been observed in a rat colon tumour model after treatment with 16,16-dimethyl-PGE_2_. Both in the normal colonic mucosa and in the tumour, a reduction of mitotic index was observed in the highest PG-dose group mediated by an increase of cAMP (Tutton and Barkla, 1980). Prostaglandin E_2_ effects in min-mice are mediated by the PG receptors EP2 and 4, which signal via cAMP. Therefore, the smaller effects we observed at the 5 *μ*M concentrations in our study may well be caused by the higher production of cAMP. However, it is difficult to assess the local tissue concentration of PGE_2_ in the *in vivo* situation so that the conclusions cannot be taken further at this point.

Single doses of PGE_2_ also induced expression of COX-2 and c-fos and stimulated signalling kinases in a time-dependent way. Maximum effects were observed after 30 min (c-fos, c-jun, signalling kinases) to 2 h (COX-2, signalling kinases). At later time points, the effects diminished as was expected from a single dose of an effector with limited chemical stability. The situation *in vivo* is much better reflected by cultures overexpressing COX-2 so that they produce less PG, but do so continually over time. In our study, this affected gene expression and signalling events in a similar manner but over at least 24 h.

In detail, addition of PGE_2_ to tumour cell cultures upregulated expression of COX-2 and consequently stimulated the endogenous production of PG in both LT97 and Caco2 cells. In LT97 cells, this event was preceeded by expression of c-fos and an increase of c-jun in the nucleus and dependent on activation of the p38 signalling kinase.

Of VEGF, a crucial PGE_2_ target gene in colonic polyps (Seno *et al*, 2002), two main splice variants were found representing the main products identified in colorectal tumour cells by Uthoff *et al* (2002). Unexpectedly, no stimulation of VEGF expression could be observed in both LT97 and Caco2 cells.

As a model for advanced adenomas VACO235 were used. These cells express high levels of COX-2 and produce as much as 1–2 nM PGE_2_ within 24 h. Their VEGF expression was higher than that of LT97 cells. As PG production could not be completely blocked by SC236, a highly specific COX-2 inhibitor (Richter *et al*, 2001), we cannot prove that the effect was PG dependent. Neither could stimulation of VEGF expression be confirmed using LT97 cells overexpressing COX-2 from an adenoviral vector. This may be due to the much smaller amount of PG produced from the transgene in LT97 cultures (250 pM). However, we cannot preclude unrelated causes. Although this seems to contradict the hypothesis that PGs act as an angiogenic switch (Seno *et al*, 2002; Sunayama *et al*, 2002), it has to be kept in mind that the adenoma cells are not the only cells capable of response to PGE_2_ present in an adenomatous polyp. In polyps *in vivo* PG can exert paracrine effects on fibroblasts, or endothelial cells, as both cell types have been shown to be responsive in other models (Gupta and Dubois, 2001; Pai *et al*, 2001; Trompezinski *et al*, 2001).

The strong expression of COX-2 in VACO235 cells also correlated with higher expression of c-fos. In this case, upregulation was confirmed by use of LT97 cells overexpressing COX-2. Inhibition by 1 *μ*M SC236 demonstrated that the effect was PG dependent. In addition, c-jun protein in the nucleus was increased in a PGE_2_-dependent manner. Induction of c-fos has also been achieved by tumour-promoting but not by non-promoting bile acids (Jurek *et al*, 2005). Stimulation of c-fos and c-jun together indicates activation of AP1 which is frequently observed following exposure to tumour-promoting compounds in the colon as well as the skin (Qiao *et al*, 2000; Angel *et al*, 2001; Bachelor and Bowden, 2004) and is regarded as a therapeutic target (Suto *et al*, 2004). Expression of c-fos was dependent on the activation of both ERK and p38 in cells exposed to PGE_2_. This indicates that more than one stimulus is necessary for induction of c-fos, which was also shown after exposure to bile acids (Qiao *et al*, 2000). In our study, both kinases were also activated in COX-2-overexpressing cells, but p38 activation could not be shown to be PG dependent.

In summary, we provide evidence that both PGE_2_ from the microenvironment and upregulation of COX-2 affect signalling and gene expression in human colorectal adenoma cells in a way that enhances tumour progression by both autocrine and paracrine mechanisms.

## Figures and Tables

**Table 1:** Polymerase chain reaction conditions for assessment of gene expression

**Gene**	**Primer sequences**	**Annealing temperatures**	**Cycles**
*GAPDH*			
Sense	5′-CGGGAAGCTTGTGATCAATGG-3′	55°C	22
Antisense	5′-GGCAGTGATGGCATGGACTG-3′		
*c-fos*			
Sense	5′-CCTGTCAAGAGCATCAGCAGCATGG-3′	60°C	29
Antisense	5′-GAGTACAGGTGACCACCGGAGTGC-3′		
*COX-2*			
Sense	5′-TTCAAATGAGATTGTGGGAAAAT-3′	55°C	32
Antisense	5′-AGATCATCTCTGCCTGAGTA-3′		
*VEGF*			
Sense	5′-CTGCTGTCTTGGGTGCATTG-3′	53°C	30
Antisense	5′-CACCGCCTCGGCTTGTCACAT-3′		

**Table 2:** TaqMan assays for quantification of gene expression

**Gene**	**Assay ID (ABI)**	**NCBI entry**	**Reporter**	**Quencher**
GAPDH	Hs99999905_m1	NM_002046	FAM	NFQ
c-fos	Hs00170630_m1	NM_005252	FAM	NFQ
COX-2	Hs00153133_m1	NM_000963	FAM	NFQ
VEGF	Hs00173626_m1	NM_003376	FAM	NFQ

**Table 3:** Relative expression VEGF in COX-2-LT97 cells

**Experimental group**	**VEGF (% of uninfected control)**
Ad-co	112.3±20.7
Ad-co+SC236	107.6±5.3
Ad-COX−2	236.7±48.7
Ad-COX−2+SC236	333.0±155.7

VEGF=vascular endothelial growth factor; COX-2=cyclooxygenase-2.
